# Cost-effectiveness of home telemonitoring in chronic kidney disease patients at different stages by a pragmatic randomized controlled trial (eNephro): rationale and study design

**DOI:** 10.1186/s12882-017-0529-2

**Published:** 2017-04-05

**Authors:** Nathalie Thilly, Jacques Chanliau, Luc Frimat, Christian Combe, Pierre Merville, Philippe Chauveau, Pierre Bataille, Raymond Azar, David Laplaud, Christian Noël, Michèle Kessler

**Affiliations:** 1 CHRU de Nancy, Plateforme d’Aide à la Recherche Clinique, Nancy, France; 2 EA 4360 APEMAC, Université de Lorraine, 54511 Vandoeuvre-lès-Nancy Cedex, Nancy, France; 3 Association Lorraine pour le Traitement de l’Insuffisance Rénale (ALTIR), Nancy, France; 4 CHRU de Nancy, Service de Néphrologie, Nancy, France; 5 CHU de Bordeaux, Service de Néphrologie Transplantation Dialyse, Bordeaux, France; 6 Université Bordeaux Segalen, Inserm U1026, Bordeaux, France; 7 Université Bordeaux Segalen, CNRS UMR5164, Bordeaux, France; 8 Université Bordeaux Segalen, Inserm U889, Bordeaux, France; 9 CH de Boulogne sur Mer, Service de Néphrologie, Boulogne sur Mer, France; 10 CH de Dunkerque, Service de Néphrologie, Dunkerque, France; 11 Société Pharmagest, Nancy, France; 12 CHU de Lille, Service de Néphrologie, Lille, France

**Keywords:** Chronic kidney disease, Cost-effectiveness evaluation, Dialysis, Home telemonitoring, Kidney transplantation, Telemedicine

## Abstract

**Background:**

Home telemonitoring has developed considerably over recent years in chronic diseases in order to improve communication between healthcare professionals and patients and to promote early detection of deteriorating health status. In the nephrology setting, home telemonitoring has been evaluated in home dialysis patients but data are scarce concerning chronic kidney disease (CKD) patients before and after renal replacement therapy. The eNephro study is designed to assess the cost effectiveness, clinical/biological impact, and patient perception of a home telemonitoring for CKD patients. Our purpose is to present the rationale, design and organisational aspects of this study.

**Methods:**

eNephro is a pragmatic randomised controlled trial, comparing home telemonitoring versus usual care in three populations of CKD patients: stage 3B/4 (*n* = 320); stage 5D CKD on dialysis (*n* = 260); stage 5 T CKD treated with transplantation (*n*= 260). Five hospitals and three not-for-profit providers managing self-care dialysis situated in three administrative regions in France are participating. The trial began in December 2015, with a scheduled 12-month inclusion period and 12 months follow-up. Outcomes include clinical and biological data (e.g. blood pressure, haemoglobin) collected from patient records, perceived health status (e.g. health related quality of life) collected from self-administered questionnaires, and health expenditure data retrieved from the French health insurance database (SNIIRAM) using a probabilistic matching procedure.

**Discussion:**

The hypothesis is that home telemonitoring enables better control of clinical and biological parameters as well as improved perceived health status. This better control should limit emergency consultations and hospitalisations leading to decreased healthcare expenditure, compensating for the financial investment due to the telemedicine system.

**Trial registration:**

This study has been registered at ClinicalTrials.gov under NCT02082093 (date of registration: February 14, 2014).

## Background

Chronic illness is the leading cause of disability and death worldwide [1], accounting for the majority of healthcare expenditures. Care for the chronically ill requires a multiplicity of providers, often resulting in fragmentation at multiple locations. Care coordination becomes an important issue since significant proportions of the population are affected. In the nephrology setting, Patients with chronic kidney disease (CKD) may remain asymptomatic at the early stages of the disease, leading to suboptimal care [2–4]. It is well documented that coordination of care between healthcare providers and multidisciplinary care teams is associated with earlier nephrology referral and reduced morbidity, mortality, overall cost and number of consultations [5]. Many countries have searched for ways to improve care coordination. Multidisciplinary care clinics have incited much interest while another possibility, telemedicine, has received relatively little attention [6]. French legislators have nevertheless recognised the potential of five telemedicine applications: teleconsultation, tele-expertise, telemonitoring, tele-assistance and coordination of medical response [7].

One promising application is home telemonitoring, a solution where active patient participation is a central component. Communication is facilitated between the patient and healthcare professionals as is interaction between healthcare providers. The characteristic feature of this mode of intervention is its capacity to strengthen patient follow-up, allowing rapid detection of early symptoms signalling potential deterioration of the patient’s health status. Theoretically, better control of chronic illness would have a significant impact on overall healthcare, limiting expenditures and reducing the need for acute care and hospitalisation. Several studies have demonstrated that telemonitoring enables better control of blood pressure, blood glucose, and asthma symptoms [8]. Telemonitoring would also improve patient autonomy by helping patients to a better understanding and control of their chronic disease and disease-related symptoms, an important incentive for treatment adherence.

Telemedicine has been evaluated in the nephrology setting for the follow-up of home dialysis patients. A clinical benefit has been demonstrated for patients on peritoneal dialysis and haemodialysis in satellite outpatient dialysis units, with a reduction in the number of hospitalisations [9–11]. For renal transplantation, data are scarce in the literature [12] and no telemedicine data are available for CKD stage 3-4 patients.

Very few studies have explored the cost-effectiveness of telemedicine for the management of chronic disease [13]. One study conducted in the United States among patients with chronic obstructive pulmonary disease and heart failure demonstrated a 4.3% reduction in mortality and a 9.8% reduction in cost [14]. To date no study has addressed the question for CKD patients.

The French national investments in the future programme initiated in 2010 included a tender designed to stimulate the development of economic models in the eHealth field (esante.gouv.fr/actus/politique-publique/). Specifically, healthcare providers were encouraged to perform studies in cooperation with industrial partners to demonstrate the usefulness of large-scale programmes offering complete service packages. Designed in this framework, eNephro was selected with its industrial promoter, Pharmagest Interactive (http://www.pharmagest.com). This background has provided a unique opportunity to evaluate the cost-effectiveness of a novel technique for home telemonitoring incorporating an expert system and centred on the active participation of CKD patients before and after renal replacement therapy.

## Methods

### Study objectives

The main objective of the eNephro study is to determine the cost-effectiveness of a telemedicine system compared with usual care, targeting blood pressure and proteinuria levels in stage 3B/4 CKD patients and 1-year duration of acute-care hospitalisation in dialysis and transplantation patients.

Secondary objectives focus on the cost-effectiveness of telemedicine to improve specific outcomes: clinical and biological parameters, perceived health status.

### Study organisation

The eNephro study is coordinated by the Nancy University Hospital. Patients are recruited in eight centres located in three administrative regions in France: 3 University Hospitals (Nancy, Lille,Bordeaux), 2 General Hospitals (Dunkerque, Boulogne sur Mer), and 3 not-for-profit healthcare providers managing self-care dialysis (peritoneal dialysis and home haemodialysis; outpatient satellite dialysis units) namely, ALTIR Lorraine, AURAD Aquitaine, and SANTELYS DIALYSE Nord Pas-de-Calais. A steering committee composed of members delegated by the eight above-mentioned centres and the industrial promoter meets quarterly to assess study progress and make decisions regarding study logistics. A scientific committee, composed of nephrologists, epidemiologists, and a health economist, works on study design, protocol and promotion. An industrial committee including a project director, an information technology director, and a clinical trial manager is in charge of project conception and coordination, information technology development and the deployment of the eNephro plateform.

### Study design and population

The eNephro study is a pragmatic randomised controlled trial (RCT) which aims to compare nephrology care delivered with a home telemonitoring system versus usual care in CKD patients at different stages of the disease. Three populations are recruited with the following inclusion criteria: age ≥ 18 years; ability to use a tablet device (alone or with assistance); population 1: stabilised stage 3B or stage 4 CKD with nephrology management of less than 3 years; population 2: stage 5D CKD treated by homecare peritoneal dialysis (PD) or out centre haemodialysis (HD); population 3: stage 5 T CKD treated by renal transplantation for 3–12 months. Non-inclusion criteria are: dialysis after renal transplantation failure; organ transplantation other than kidney; life expectancy < 1 year.

### Patient census and enrolment

**Fig. 1:**
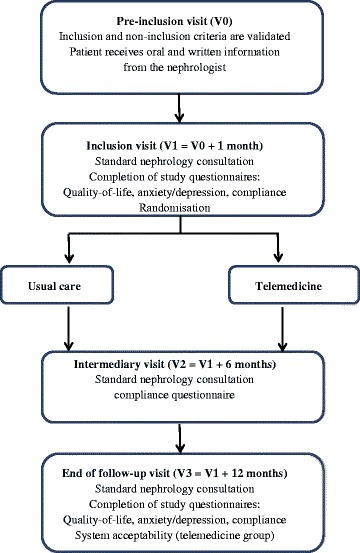
Flow-chart of the eNephro study

During the 24-month inclusion period, participating centres are invited to enrol eligible patients attending a pre-inclusion medical visit (V0) where inclusion and non-inclusion criteria are validated (Fig. 1). An inclusion visit (V1) is conducted one month later as a standard nephrology consultation with a physical examination, blood and urine tests according to guidelines of the French health authorities (HAS) [15–17], and therapeutic adjustment as needed.

At the end of the inclusion visit, eligible patients are informed about the eNephro study and asked to sign an informed consent form. Patients who agree to participate in the study are randomly assigned to a study group (intervention or control) using a random number generator integrated in the telemedicine system. Randomisation is performed by balanced blocks of 20 and stratified by -participating centre and population (1, 2, or 3). Participating patients are then referred to a clinical research nurse who collects pertinent data (see data collection below) and assists patients in filling out the study questionnaires (see outcomes of interest below). Patients assigned to the intervention group are given a commercially available 10-inch tablet device with instructions for use of the telemedicine patient interface.

### Patient follow-up

The follow-up period is set at 12 months. Patients are scheduled for an intermediary visit at six months (V2) and a final end-of-study visit at 12 months (V3), conducted with the same protocol as the inclusion visit (V1). Patients in the intervention group are asked to respond to a questionnaire on the acceptability of the telemedicine system in addition to the other questionnaires (Fig. 1).

The nephrology care schedule for patients in populations 1 and 2 abides by the French recommendations [10, 15, 17], likewise for transplanted patients (population 3) in the control group. For transplanted patients in the telemonitoring group, considering the number of visits regularly scheduled during the first post-transplantation year [17], the Medical team has the possibility of cancelling every other in centre visit when clinical and biological results, transmitted by the patient are in the target and no symptom declared. Each patient’s telemedicine system transmits the following information to the data platform: body weight; body temperature; pulse rate; patient-measured systolic and diastolic blood pressure for the last three days; patient-assessed presence of symptoms including vertigo, short breath, fatigue, oedema, abdominal pain, deterioration of general health status. The telemedicine system also transmits to the data platform information issuing from medical laboratories, e.g. the biological parameters recommended by the French authorities (HAS) for the follow-up of kidney transplant recipients on samples taken three days before scheduled visits.

### Description of the eNephro telemedicine system

eNephro is an eHealth information technology specifically developed for the care of CKD patients at all stages of the disease. This web-based application, harboured by secure servers according to French regulations, ensures the security, confidentiality, integrity, sustainability, availability, reversibility and tracability of collected data. As a web-based application, eNephro is accessible from any web browser.

**Fig. 2:**
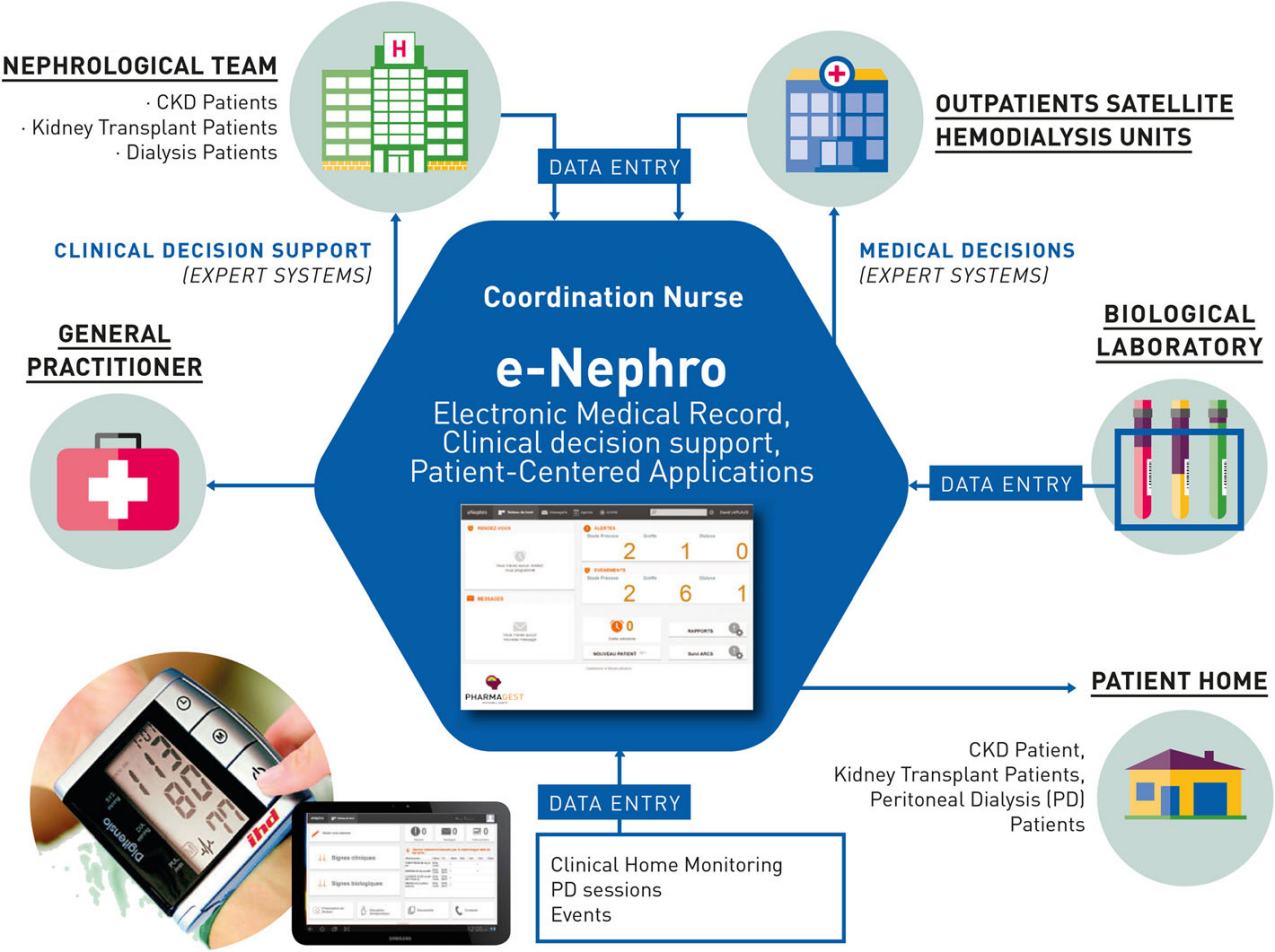
The eNephro system

The information system is composed of a shared electronic medical record, a secure messaging application used for communication between healthcare professionals and patients, an agenda for scheduling medical visits, and a telesurveillance application specifically designed for each population studied. In addition, eNephro harbours several expert systems, e.g. clinical decision support tools, for analysing patient data (Fig. 2).

eNephro’s expert systems issued from collaboration between the Lorraine Research Laboratory in Computer Science and its Applications (LORIA^1^) and nephrologists. These expert systems have been used since 2000 by end-stage renal disease (ESRD) patients on home dialysis [18–21], and 2006 by kidney transplant recipients [12]. An expert system for stage 3B/4 CKD patients was specifically developed for the purpose of this study.

eNephro’s telemonitoring system enables remote collection of clinical parameters measured by the patient and biological parameters determined by medical laboratories. The expert systems perform an automatic analysis of collected data on a daily basis. This automatic analysis filters the large data mass corresponding to normal situations not requiring any particular action and records charts to be examined at the next scheduled visit. A clinical decision support algorithm modelling medical reasoning is included in the system to detect situations of risk.

These expert systems are based on a Bayesian approach [22, 23]. An alert is generated when the analysis of the clinical and biological data indicates with high probability an alteration of the patient’s health status, before the development of clinical manifestations. The alerts generated by the expert systems are designed for early detection of the following situations: risk of dehydration/hyperhydration, poor blood pressure control, elevated proteinuria, poor anaemia control, occurrence of a complication, recognised with predefined symptoms.

Users (patients and healthcare professionals) connect via their user-dedicated interface secured with a strong authentication device: Public Key Infrastructure. Healthcare professionals use their own computer to access eNephro and patients are equipped with a tablet device.

#### Patient interface

Patients can access information concerning their personal situation. Patient interface functions are described in Table 1.

**Table 1:** eNephro patient interface

Function	Patient input	Patients have access to:
Messages	Messages for care team	Messages from the care team
Agenda		Visit schedule
Recalls		Recalls concerning data collection and/or planned visits
Contacts		Medical team, clinical research nurses
Documents	Information concerning care received outside the nephrology setting	Medical documents shared by the care team
Medicinal treatment		Latest prescription written by the nephrologist
Patient data		
	Dialysis patient: Data on HD or PD sessions	Prescription for dialysis History of HD or PD sessions
	CKD patient: Clinical home monitoring (blood pressure, weight dyspnoea, oedema, fatigue) Data collection: every 2 weeks	Time course graphs of clinical parameter values History of data collection (function: clinical symptoms)
	Renal transplant patient: Clinical home monitoring (blood pressure, weight dyspnoea, oedema, fatigue) Data collection: from every week to every month depending on time since transplantation	
Events	All patients: Any change in health status or medicinal treatment	
Clinical symptoms		Time course graphs of clinical parameter values
Laboratory results		Laboratory test results reported by the Clinical Research Nurse

#### Health practitioner interface

Daily surveillance is ensured by a coordinating nurse who processes incoming data and alerts, handles aspects within his/her competency and transfers other information to the centre’s referral nephrologist. The referral nephrologist builds up patients’ medical records, manages the enrolment process, and performs intermediary and final follow-up visits. The referral nephrologist is also responsible for recording events occurring during the follow-up period, managing daily alerts transmitted by the coordinating nurse, and responding to patient messages. The patient’s general practitioner can consult the patient’s medical record at any time. Medical laboratories transfer biological results directly to the data processing platform.

### Data collection

#### * Data recorded at inclusion and during follow-up

An electronic case report form (eCRF) is used to collect socio-demographic, clinical, biological and therapeutic data from patient records at specified time intervals in the three patient populations (Table 2).

**Table 2:** Data recorded at inclusion and during follow-up

Category	Data collected	Inclusion	6 months	12 months
Sociodemographic data	Age, sex, residence (French administrative district), social-occupational category [32] Educational level, housing, marital status	X		
Clinical data	CKD history	X		
	Medical history and risk factors	X		
	General health status, weight, estimated dry weight (population 2), blood pressure, other clinical measurements	X	X	X
	Participation in a patient education programme within the last six months	X	X	X
	Status of the vascular access or the peritoneal catheter (population 2)	X	X	X
	Intercurrent events^a^ occurring within the last six months		X	X
Biological data	Laboratory test results as recommended by the French health authorities (HAS) [15, 17]	X	X	X
Therapeutic data	Dialysis history (populations 2 and 3)	X		
	Transplantation history (population 3)	X		
	Dialysis prescriptions (population 2)	X	X	X
	Medications (drug names, dosages)	X	X	X

^a^Intercurrent events: major cardiovascular and/or nephrological events, hospitalisations, death, rejection of the telemedicine system

Study completion is noted as are patient dropouts together with the reason (lost to follow-up, medical decision, consent withdrawal, change in disease stage, death). All data are collected and checked for completeness by nine trained clinical research nurses. An independent clinical research nurse compares 10% of the completed eCRFs for consistency with the patient’s medical record.

#### * Economics data

Cost values correspond to the tariffs set by the French healthcare fund. Only direct costs are taken into account for the cost analysis. Two types of cost are considered: 1) intervention-related cost (computer equipment, maintenance, personnel training for the telemedicine system), recovered from the telemedicine system supplier; 2) patient management-related costs (hospitalisation, consultation, home visits by nurses, complementary explorations, drugs and medical devices, health transport). This part of the economics data stems from the French health insurance database SNIIRAM^2^ that contains individualised, anonymous and linkable data. Prospectively recorded for all beneficiaries of healthcare in France, SNIIRAM covers the entire French population (66 million inhabitants). Data recorded include all medical expenditure reimbursements for long-term disabling diseases, e.g. CKD. A probabilistic data linkage method [24] will be applied to identify patients included in the eNephro study within the anonymous SNIIRAM database.

For each population, mean cost per patient and mean cost per group (intervention vs control) are estimated, allowing to calculate an Incremental Cost Effectiveness Ratio (ICER) of telemedicine as compared with usual care. The ICER relates the overall costs to the primary endpoint for each population.

### Outcomes of interest

Outcomes of interest are presented in Table 3.

**Table 3:** Outcomes of interest in the e-nephro study

Populations	Outcomes of interest
Primary outcome of interest	
Population 1	Cost-result ratio, where result is achievement of blood pressure (systolic ≤ 130 and diastolic ≤ 80 mmHg) and proteinuria (≤0.5 g/24 h or proteinuria/creatininuria ≤ 50 mg/mmol) recommended targets at V3 [33].
Populations 2 and 3	Cost-result ratio, where result is the cumulative number of days of acute-care hospitalisation over the 1-year study period (from V1 to V3).
Secondary outcomes of interest	
Populations 1, 2, and 3	Medication compliance measured by the French questionnaire by Girerd [34]
	Anxiety/depression level, measured with the Hospitalization Anxiety Depression Scale (HADS) [35, 36]
	Quality-of-life, measured with the KDQoL V36 questionnaire [37] for populations 1, 2 and the ReTransQoL questionnaire [38] for population 3
Intervention group of populations 1, 2, and 3 + healthcare practio-ners	Acceptability of the telemedicine system, measured with the French version of the Service User Technology Acceptability Questionnaire (WSD SUTAQ) for telemonitoring projects [39, 40]. The patient dropout rate from the telemedicine system will also be considered an indicator of the patient acceptability.
Populations 1 and 3	1-year change in glomerular filtration rate measured with the MDRD formula [41]
Population 3	Number of planned nephrology outpatient visits actually performed, and the number of unplanned visits and hospitalisations over the 1-year study period
Population 2	Event-free survival at 1 year, an event being defined here as return to in-centre dialysis or hospitalisation
	Anaemia control, defined as achievement of haemoglobin, ferritin and coefficient of transferrin saturation recommended targets

### Statistical analyses and sample size

Data processing and statistical analysis are performed by the clinical epidemiology centre of Nancy using SAS® software version 9.5 (SAS Institute, Inc., Cary, N.C.).

#### * Main data analyses

The statistical analysis plan includes the following procedures for each of the three study populations:

(i) A description of the patients included in each group (intervention, control): number of patients, socio-demographic, clinical, biological and therapeutic parameters. To check the comparability of patient groups, the main features are compared using the *χ* ^2^ test for qualitative variables, analysis of variance or the Mann-Whitney test for quantitative variables.
(ii) A description of deviations from the protocol.
(iii) Inter-group comparison of clinical outcomes and cost-result ratios, with adjustment for any characteristic differing between two groups. Intention-to-treat analysis is applied. The percentage of patients in population 1 who reached blood pressure and proteinuria targets is compared between the intervention and control groups using a logistic regression model. For populations 2 and 3, mean durations of acute-care hospitalisation and cost-result ratios are compared between the intervention and control groups using linear regression models.

#### * Sample size

Sample size calculations were made to demonstrate superiority based on the estimated proportions/mean difference of the primary outcome of interest between groups (telemedicine versus usual care). The alpha risk was set at 5% and the beta risk at 10%. The total number of patients to include is 320 for population 1 (difference of 15% in the primary outcome of interest between groups), and 260 for populations 2 and 3 (mean difference: 6 days, standard deviation: 15 days for population 2 and mean difference: 8 days, standard deviation: 20 days for population 3).

## Discussion

There have been very few assessments of the medicoeconomic interest of home telemonitoring for chronic diseases, and the quality of evidence has often been weakened by methodological flaws. Therefore, it is difficult to compare home telemonitoring with usual care for the chronic diseases studied [25]. eNephro is the first medicoeconomic study designed to evaluate the benefits of home telemonitoring for CKD patients at different stages. The implementation of telemedicine applications differs from other health interventions, such as the use of medications or devices, in that these applications contain several interacting components and have effects that are highly dependent on the local context in which they take place. Evaluating such interventions, qualified as complex interventions [26], raises practical and methodological issues that researchers have to take into account. Guidance for developing and evaluating complex interventions was published by the Medical Research Council in 2008 [26]. Later, national and international templates for the specific evaluation of telemedicine projects, integrating general recommendations about the assessment of complex interventions, were published in 2012-13 (HAS [27], MAST [28]). All templates emphasize the need for a comparative design [29], but acknowledge the trade-off between methodological requirements to ensure internal validity and the necessity to permit a degree of flexibility or tailoring of the assessed intervention. Ensuring strict fidelity to a protocol (e.g. medications trials) may be inappropriate as the intervention may work better if adaptation to the local setting is allowed. The choice of a pragmatic trial for our evaluation combines the methodological requirements (comparative design and randomisation) and the necessary flexibility in the implementation of the intervention. Templates also underscore the usefulness of multiple outcomes and the integration of patients’ and professionals’ points of view, important aspects included in the eNephro evaluation. Moreover, the effects of telemedicine on patient health – clinical parameters, morbidity, mortality, health-related quality-of-life – are evaluated and all resources used are considered in the cost evaluation, according to the above-mentioned recommendations. The French health insurance database (SNIIRAM) appears to offer the best way to estimate medical expenditures incurred for a given patient during a given period. SNIIRAM records data for all healthcare beneficiaries in France, and includes the reimbursed costs of all out-of-hospital healthcare consumptions, as well as all hospital stays and consultations in public or private institutions [30]. Finally, the acceptability of the telemedicine system and satisfaction with its use are assessed, as recommended, for all stakeholders, i.e. patients randomised to intervention groups and health professionals involved in the home telemonitoring.

Many types of results are expected to come forward. For patients with stage 3B/4 CKD, eNephro home telemonitoring should allow enhanced follow-up since clinical and biological information will be recorded regularly by the data platform. It is also expected that the expert systems identifies early situations where patients risk for decompensation and/or deterioration of their health status, even before the development of clinical symptoms prompt them to seek medical assistance. This should enable more rapid medical intervention and a lower rate of hospitalisation. Better control of blood pressure and proteinuria should delay the need for more aggressive care with replacement therapy. For patients on home and out centre dialysis (haemodialysis or peritoneal dialysis), regular transmission of data issuing from various sources (patient, health practitioners, medical devices, medical laboratories) should lead to earlier and more rapid care for complications, with a reduction in the need for hospitalisation, an important source of medical expenditure in this population. It has been demonstrated that an expert system evaluating the state of hydration of patients on peritoneal dialysis enables a considerable reduction in the number of hospitalisations for acute pulmonary oedema [19]. Furthermore, knowing one is under telesurveillance and that important health data can be transmitted quickly is reassuring. For patients on haemodialysis, the transmission of data issuing from the generator during the dialysis session improves the detection of problems involving a malaise [31] or the vascular access [18]. Transplant recipients should also benefit from telesurveillance facilitated by a specifically designed expert system [12], and the number of visits to the transplantation centre may be limited when clinical and biological parameters remain within target ranges.

Use of a home telemonitoring system affects medical routine practices, implying to adapt and reorganise the physicians work schedule. It’s necessary to fix daily time slots for monitoring alerts, communicating with patients, or adapting treatments. As the mass of data generated by the system is significant, a telemedicine coordinator (e.g. a coordinating nurse) seems to be necessary to deal with all information transmitted. The reduction in the need for in-centre visits undoubtedly releases time for this new allotment. The acceptability of a telemedicine system by physicians depends heavily upon the capacity for optimal reorganisation, and the realisation that home telemonitoring activity is not simply an added work load. The clinical situations requiring management should be less complicated, and thus less time demanding since detected earlier by the expert system. Home telemonitoring also facilitates communication and cooperation between specialists, disciplines, and referral levels (nephrology – 2^nd^ line referral, general medicine – 1^st^ line referral), undoubtedly leading to improved patient-centred care. All of these elements join together to facilitate the delivery of higher quality care, a common goal for patients, professionals and healthcare systems.

For the healthcare system, home telemonitoring also contributes to cost containment by limiting the need for patient transportation, by reducing the number of in-centre visits, hospitalisations and by favouring early care for deteriorated health status and by preventing complications. In the eNephro study, it is expected that the costs generated by the telemedicine system are counterbalanced by lower overall healthcare costs. In this case, the results would be an important argument favouring reimbursement of home telemonitoring costs by the national health insurance fund.

This study can be expected to identify barriers and facilitators for implementing a home telemonitoring system as well as profiles of patients most likely to benefit from this new care system.

The first patient was enrolled in the eNephro study on November 27, 2015. At the time this article was submitted, a total of 259 patients (127 telemedicine patients; 132 control patients) had been enrolled.

## Abbreviations

ALTIR: Association lorraine pour le traitement de l’insuffisance rénale
AURAD: Association pour l’utilisation du rein artificiel
CKD: Chronic kidney disease
e-CRF: Electronic case report form
ESRD: End stage renal disease
HAS: Haute autorité en santé
HD: Haemodialysis ICER, incremental cost-effectiveness ratio
MAST: Model for assessment of telemedicine
PD: Peritoneal dialysis
RCT: Randomised controlled trial
SNIIRAM: Système national d’information inter-régimes de l’assurance maladie.

## Footnotes

1 LORIA : *Laboratoire Lorraine de Recherche en Informatique et ses Applications*

2 SNIIRAM : *Système national d’information inter-régimes de l’assurance maladie*

## References

[1] World Health Organization. Global Status Report on Noncommunicable Diseases. 2014. http://apps.who.int/iris/bitstream/10665/148114/1/9789241564854_eng.pdf?ua=1. Accessed 10 Nov 2016.

[2] Hallan SI, Coresh J, Astor BC, Asberg A, Powe NR, Romundstad S (2006). International comparison of the relationship of chronic kidney disease prevalence and ESRD risk. J Am Soc Nephrol.

[3] REIN. Rapport Annuel. 2013. http://www.agence-biomedecine.fr/IMG/pdf/rapport_rein2013.pdf. Accessed 10 Nov 2016.

[4] Van Gelder VA, Scherpbier De Haan ND, De Grauw WJ, Vervoort GM, Van Weel C, Biermans MC (2016). Quality of chronic kidney disease management in primary care: a retrospective study. Scand J Prim Health Care.

[5] Wang SM, Hsiao LC, Ting IW, Yu TM, Liang CC, Kuo HL (2015). Multidisciplinary care in patients with chronic kidney disease: a systematic review and meta-analysis. Eur J Intern Med.

[6] Gordon EJ, Fink JC, Fischer MJ (2013). Telenephrology: a novel approach to improve coordinated and collaborative care for chronic kidney disease. Nephrol Dial Transplant.

[7] Hospital, patients, Health and Territory. Edited by Health Ministry, vol. Decree No. 2010–1229 Art.R. 6316–1 of 19 October 2010. 2009. https://www.legifrance.gouv.fr/affichTexte.do?cidTexte=JORFTEXT000020879475&categorieLien=id. Accessed 10 Nov 2016.

[8] Pare G, Moqadem K, Pineau G, St Hilaire C (2010). Clinical effects of home telemonitoring in the context of diabetes, asthma, heart failure and hypertension: a systematic review. J Med Internet Res.

[9] Gallar P, Vigil A, Rodriguez I, Ortega O, Gutierrez M, Hurtado J (2007). Two-year experience with telemedicine in the follow-up of patients in home peritoneal dialysis. J Telemed Telecare.

[10] HAS. Les conditions de mise en œuvre de la télémédecine en unité de dialyse médicalisée. 2010. http://www.has-sante.fr/portail/upload/docs/application/pdf/2010-01/synthese_conditions_telemedecine_udm_vf.pdf. Accessed 10 Nov 2016.

[11] Nakamoto H (2007). Telemedicine system for patients on continuous ambulatory peritoneal dialysis. Perit Dial Int.

[12] Kessler M, Erpelding ML, Empereur F, Rose C (2013). Transplantelic: an application of telemedicine in kidney transplantation. Eur Res Telemed.

[13] Wootton R (2012). Twenty years of telemedicine in chronic disease management--an evidence synthesis. J Telemed Telecare.

[14] Baker LC, Johnson SJ, Macaulay D, Birnbaum H (2011). Integrated telehealth and care management program for Medicare beneficiaries with chronic disease linked to savings. Health Aff.

[15] HAS. Guide du Parcours de Soins - Maladie Rénale Chronique de l'Adulte. 2012. http://www.has-sante.fr/portail/upload/docs/application/pdf/2012-04/guide_parcours_de_soins_mrc_web.pdf. Accessed 10 Nov 2016.

[16] HAS. Qualité de la prise en charge des patients hémodialysés chroniques, IPAQSS 2015. 2015. http://www.has-sante.fr/portail/upload/docs/application/pdf/2015-11/synthese_dia_2015_vd.pdf. Accessed 10 Nov 2016.

[17] HAS. Suivi ambulatoire de l’adulte transplanté rénal au-delà de 3 mois après transplantation. Recommandations professionnelles. 2007. http://www.has-sante.fr/portail/upload/docs/application/pdf/suivi_du_transplante_renal_-_synthese_des_recommandations.pdf. Accessed 10 Nov 2016.

[18] Chanliau J, Charasse C, Rose C, Bene B (2014). Clinical evaluation of an expert system for arteriovenous fistula assessment. Int J Artif Organs.

[19] Durand PY, Chanliau J, Kessler M, Romary L, Thomesse JP, Charpillet F (2000). DIATELIC: A new intelligent telemedicine system to avoid hdration disorders in CAPD patients. Perit Dial Int.

[20] Durand PY, Chanliau J, Mariot A, Thomesse JP, Romary L, Charpillet F (2001). Cost-benefit assessment of a smart telemedicine system in patients undergoing CAPD: Preliminary results. Perit Dial Int.

[21] Rose C, Smaili C, Charpillet F (2005). A dynamic Bayesian network for handling uncertainty in a decision support system adapted to the monitoring of patients treated by hemodialysis. 17th IEEE international conference on tools with artificial intelligence - ICTAI'05: 2005-11-14 2005; Hong-Kong: IEEE computer society Washington, DC, USA ©2005.

[22] Pearl J (1988). Probalistic reasoning in intelligent systems: networks of plausible inference.

[23] Rabiner LR (1989). A tutorial on hidden Markov models and selected applications in speech recognition. Proc IEEE.

[24] Silveira DP, Artmann E (2009). Accuracy of probabilistic record linkage applied to health databases: systematic review. Rev Saude Publica.

[25] Campbell NC, Murray E, Darbyshire J, Emery J, Farmer A, Griffiths F (2007). Designing and evaluating complex interventions to improve health care. BMJ.

[26] Craig P, Dieppe P, Macintyre S, Michie S, Nazareth I, Petticrew M (2008). Developing and evaluating complex interventions: the new Medical Research Council guidance. BMJ.

[27] HAS. Efficience de la télémédecine : état des lieux de la littérature internationale et cadre d’évaluation, rapport d’évaluation médico-économique. 2013. www.has-sante.fr/portail/upload/docs/application/pdf/2013-07/efficience_tlm_vf_2013-07-18_14-48-38_743.pdf. Accessed 10 Nov 2016.

[28] Kidholm K, Ekeland AG, Jensen LK, Rasmussen J, Pedersen CD, Bowes A (2012). A model for assessment of telemedicine applications: mast. Int J Technol Assess Health Care.

[29] Mistry H, Garnvwa H, Oppong R (2014). Critical appraisal of published systematic reviews assessing the cost-effectiveness of telemedicine studies. Telemed J E Health.

[30] Moulis G, Lapeyre Mestre M, Palmaro A, Pugnet G, Montastruc JL, Sailler L (2015). French health insurance databases: what interest for medical research?. Rev Med Interne.

[31] Tangaro S, Fanizzi A, Amoroso N, Corciulo R, Garuccio E, Gesualdo L (2016). Computer aided detection system for prediction of the malaise during hemodialysis. Comput Math Methods Med.

[32] INSEE. Nomenclature des professions et categories socio-professionnelles. 2003. http://www.insee.fr/fr/methodes/?page=nomenclatures/pcs2003/pcs2003.htm. Accessed 10 Nov 2016.

[33] ANAES. Moyens Thérapeutiques pour ralentir la progression de l’Insuffisance Rénale Chronique chez l'adulte. Recommandations pour la pratique clinique. 2004. http://www.has-sante.fr/portail/upload/docs/application/pdf/IRC_2006_recos.pdf. Accessed 10 Nov 2016.

[34] Girerd X, Hanon O, Anagnostopoulos K, Ciupek C, Mourad JJ, Consoli S (2001). Assessment of antihypertensive compliance using a self-administered questionnaire: development and use in a hypertension clinic. Presse Med.

[35] Snaith RP (2003). The hospital anxiety and depression scale. Health Qual Life Outcomes.

[36] Zigmond AS, Snaith RP (1983). The hospital anxiety and depression scale. Acta Psychiatr Scand.

[37] Boini S, Bloch J, Briancon S (2009). Monitoring the quality of life of end-stage renal disease patients. Quality of life report - REIN - dialysis 2005. Nephrol Ther.

[38] Gentile S, Jouve E, Dussol B, Moal V, Berland Y, Sambuc R (2008). Development and validation of a French patient-based health-related quality of life instrument in kidney transplant: the ReTransQoL. Health Qual Life Outcomes.

[39] Charrier N, Zarca K, Durand Zaleski I, Calinaud C, Group ARSIDFT (2016). Efficacy and cost effectiveness of telemedicine for improving access to care in the Paris region: study protocols for eight trials. BMC Health Serv Res.

[40] Hirani SP, Rixon L, Beynon M, Cartwright M, Cleanthous S, Selva A, Sanders C, Newman SP; WSD investigators. Quantifying beliefs regarding telehealth: Development of the Whole Systems Demonstrator Service User Technology Acceptability Questionnaire. J Telemed Telecare. 2016. [Epub ahead of print]. 10.1177/1357633X16649531 27224997

[41] Levey AS, Coresh J, Balk E, Kausz AT, Levin A, Steffes MW (2003). National Kidney Foundation practice guidelines for chronic kidney disease: evaluation, classification, and stratification. Ann Intern Med.

